# Equitable spam detection and prevention in a community-engaged public health survey

**DOI:** 10.3389/fpubh.2026.1751698

**Published:** 2026-10-13

**Authors:** Sarah Farash, Holly Sienkiewicz, Marc Solomon, Kathi Lynch

**Affiliations:** Common Ground Health, Rochester, NY, United States

**Keywords:** data integrity, equity, online survey, public health, spam detection

## Abstract

My Health Story is a community-engaged, public health survey administered to adults aged 18 and older in the Finger Lakes region of New York state by a community-based planning, research, and convening agency. In 2022, shortly after initiating recruitment, the team noticed that the survey database was receiving an unusually high rate of responses from links posted online. After scanning these records, the team questioned whether many were inauthentic, or “fake” data responses. In response, study investigators created an *ad hoc* data equity team to engage in a self-reflective, iterative process to develop and test strategies for detecting and preventing further infiltration of inauthentic records. Importantly, the study team engaged in processes to double check that flag definitions did not unfairly exclude underrepresented populations, the very same people that the project was designed to recruit. Ultimately, the effort preserved the integrity of the dataset while prioritizing the inclusion of underrepresented populations. We conclude with recommendations for equitable prevention and detection of inauthentic records and with regulatory considerations for similar public health survey initiatives.

## 1 Background

In New York State, primary data collection is a key component of county public health departments’ Community Health Assessment process and is often fulfilled by conducting anonymous, public health surveys. Many public health departments rely on internet-based recruitment strategies to reach their respective audiences. While web-based recruitment strategies are a convenient method for gathering responses, malicious actors can infiltrate public health surveys with inauthentic responses threatening the integrity of survey results (1–3). Additionally, recommended practices for prevention and identification of inauthentic records do not account for potential biases to inclusion of underrepresented research populations introduced by logic check-based record flagging algorithms (4, 5). Public health researchers using public-facing, internet-based recruitment methods need to be aware of this phenomenon and plan ahead to protect the inclusion of underrepresented populations amidst ongoing, evolving spam infiltration attempts.

My Health Story is a community-engaged, public health survey. We present a retroactive review of our unanticipated journey of preventing and detecting inauthentic records while prioritizing equity and the representation of traditionally marginalized populations in our work (6). Data collected from the My Health Story project supports the community health assessment process for multiple counties in the Finger Lakes region of New York state. My Health Story participant recruitment methods were designed to increase inclusion of historically marginalized populations’ experiences of social drivers of health and self-reported health statuses. The 2022 iteration of My Health Story survey (MHS 2022) was the first time project researchers encountered inauthentic record infiltration. In this report, the terms ‘inauthentic record,’ ‘spam,’ or ‘fake data’ are used interchangeably to mean completed survey records input by a computer program or people who were knowingly misrepresenting themselves in their answer choices. This definition is similar across multiple case studies of similar phenomena (5, 7, 8).

Had we not recognized the inauthentic infiltration of records, our resulting sample would have appeared significantly younger with disproportionately high rates of inclusion from low-population density ZIP codes in the region. Additionally, the 8-month long process recognizing, mitigating, and detecting inauthentic records meant we could not effectively track our recruitment progress in real time, nor could we adequately adjust recruitment efforts to meet the goals of our quota sampling plan. Ultimately, we ended up with a usable dataset due to iterative reflection processes that we engaged in with our research team and input from our community-engaged staff members.

Equity is generally defined as “fairness or justice in the way people are treated.” (9) We further define ‘equity in detection and prevention of spam’ as a process of explicitly examining all methods and strategies to mitigate the chance of unfairly removing a record from our dataset. To assist future public health researchers to equitably design infiltration prevention and detection strategies before recruitment begins, we begin with a detailed look into our experience with inauthentic record discovery by providing two examples highlighting differences in outcomes that would have occurred had we not discovered the infiltration and continued to use the fully collected dataset. We then illustrate implications that the phenomenon of inauthentic record infiltration has for the informed consent process and risk-to-benefit assessment of human subject research projects. After detailing the significance of the problem and the regulatory requirement implications for future projects, we present a summary of our iterative process that emphasized minimizing the potential for exclusion of possibly legitimate records from traditionally underrepresented populations. We conclude with a summary of recommendations for future public health researchers.

## 2 Infiltration significance and research ethics implications

To gauge the impact that undiscovered inauthentic record infiltration would have had on our project, we did a *post hoc* analysis to see how the inauthentic records sample differed from the authentic dataset that excluded all suspicious records. This was an important step for our agency to justify utilizing 8-months of staff time and physical resources to ensure that the final My Health Story 2022 dataset accurately reflected the perspectives of participants in our region. We begin this report by presenting post-hoc analyses to show that spam prevention and detection efforts result in meaningful differences compared to if these efforts had not been carried out.

Table 1 shows the age distribution of survey respondents from Monroe County, NY where we heavily recruited in-person and online for MHS 2022. The table shows basic descriptive statistics for responses to the write-in question, “How old are you?” for respondents who also selected that they lived in this particular county. Notably, we utilized an *a priori* data validation tool to prevent respondents who are less than age 18 or older than age 100 from entering answers. Thus, traditional data cleaning strategies, such as weeding out outliers, would not have been enough to find and remove inauthentic survey data using this question.

**Table 1:** Independent samples *t*-test for age demographic in the My Health Story 2022 dataset.

My Health Story 2022	Mean	Std Dev	95% confidence interval		*N*
Inauthentic records	32.2^*^	9.2	31.7	32.6	1473
Authentic records	49.6^*^	18.3	48.8	50.3	2151

^*^p < 0.0001. Data excerpt represents a single county’s respondents only.

Table 1 details the statistically significant t-test result comparing the mean ages of inauthentic and authentic records [*t*(3351) = −37.7, *p* = <0.0001]. The inauthentic records average age (M = 32.2, SD = 9.2) was younger with a tighter distribution of data around the mean than the authentic records average age (M = 49.6, SD = 18.3.) Our results would have yielded inaccurate conclusions muddied by fake data records that were significantly younger than records without suspicious characteristics. Had we not recognized the infiltration of inauthentic records and worked diligently to prevent, find, and remove them, using this dataset to inform older adult programming and policy in Monroe County, NY would have been inaccurate and misleading.

To demonstrate evidence of the severe disproportion in the geographic distribution of respondents that we received compared to our expectations, Figure 1 shows records for Chemung County, NY by ZIP code in the MHS 2022 dataset compared to expected population proportions. The map on the right illustrates the sample rate by ZIP code for survey takers noting that they lived in the county, while the map on the left illustrates expected population proportions by ZIP code using in-house population count estimates for adults aged 18 and older (10).

**Figure 1:**
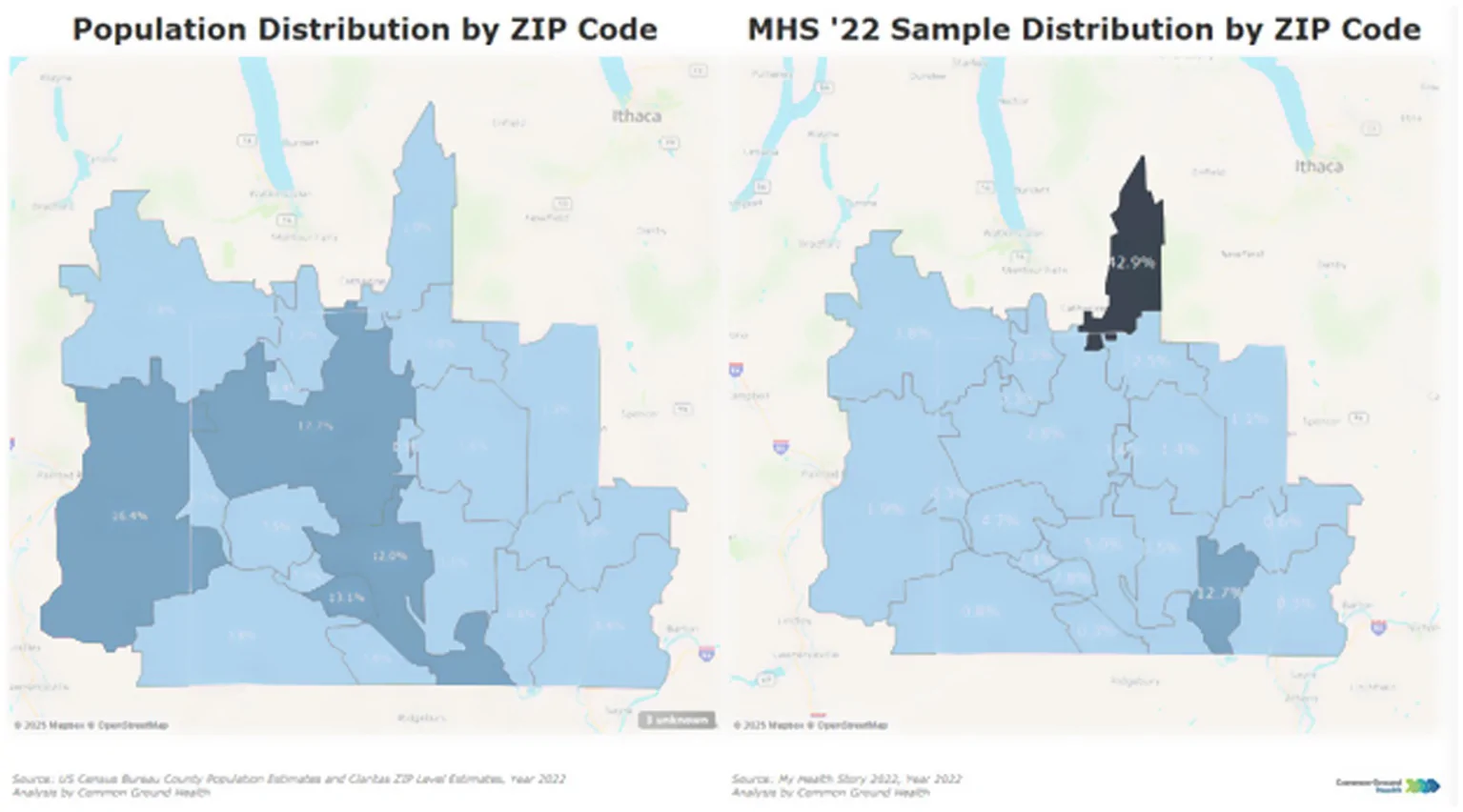
Chemung County population and My Health Story 2022 sample distributions by ZIP code.

Had we continued to use the dataset without questioning the integrity of the data, our resulting dataset would have had severely disproportionate representation from low-density ZIP codes, such as in the northernmost ZIP code area in Figure 1 which represented 43% of the sample dataset but only 1% of the population in Chemung County, NY. We present these data points as evidence to emphasize the magnitude and importance of this issue in public health research. Public health officials and those relying on publicly disseminated recruitment methods must increase their awareness of this phenomenon and plan ahead to address the ongoing, technologically evolving capacities that inauthentic survey takers employ to ensure that their findings are not accidentally misconstrued by falsified data records.

In addition to threats to data integrity, there are human subjects research ethics considerations resulting from inauthentic record infiltration. Our project qualified for expedited review. According to Section 46.111(a)(2) of federal regulation policy 45 CFR 46 governing human subjects research, expedited research projects must meet criteria ensuring that the “Risks to subjects are reasonable in relation to anticipated benefits…and the importance of the knowledge that may reasonably be expected to result.” (11) As demonstrated using data from MHS 2022, inauthentic record infiltration directly impacts the validity and integrity of the knowledge produced by research projects. Although the potential threat of inauthentic record infiltration does not directly impact the research experiences of authentic survey participants, this phenomenon increases the risk that survey participants’ time taking the survey is wasted and that their survey responses may ultimately be unusable should spam detection efforts fail.

Online survey research project investigators should be required to mention in the informed consent or background information sections the risk that research participants’ survey data might be unused if the project is infiltrated by inauthentic records and researchers are unable to decipher legitimate from fake records. We provide suggested language to include in background information sections of online public health research projects that helps researchers transparently explain the risks associated with potential inauthentic record infiltration. In the following sections, we detail our efforts including underrepresented populations while spontaneously creating and modifying our prevention and detection strategies.

### 2.1 Overview of survey purpose and methods

My Health Story 2022 was designed to support our agency’s health equity mission and local public health departments’ community health assessment processes. The survey was intended to include residents aged 18 and older, from a 12-county catchment area in the Finger Lakes region and Western New York. The survey was developed and tested in collaboration with over 100 local communities and non-profit agencies focused on improving health outcomes and health equity among residents in the region. Data was collected in an online version of the survey using REDCap, a private, server-hosted software (12, 13). Originally, all survey takers received a small, monetary compensation for their time. This information was included in our print and electronic recruitment materials. Data equity principles, such as inclusion, accessibility, and transparency were referenced and operationalized throughout survey development and in planning our recruitment efforts (14). Biomedical Research Alliance of New York Institutional Review Board provided regulatory oversight for the project (15).

Our survey design and initial recruitment plan prioritized oversampling historically marginalized populations including people of color, Indigenous people, new Americans, and people who speak languages other than English to take the survey. In agreement with known strategies to increase recruitment of less-often included populations, respondents were recruited using non-probability, convenience and snowball sampling through research-trained, community ambassadors (16–18). Research ambassadors attended a mix of in-person, community events in the survey catchment region. They handed out post-cards within a robust network of faith-based congregations, barber shops, and beauty salon sites and utilized mobile technology to create internet hot-spots for touch screen devices to allow people to complete the survey on-the-spot, even in areas with unstable or inaccessible internet connectivity. All recruitment materials had QR codes and TinyURL (19) tracking website links associated with specific REDCap survey links. Identifying records collected in-person and through specific types of online recruitment activities was integral to our prevention and detection efforts.

### 2.2 Admitting we have a problem

Less than 1 week after recruitment opened, we saw a large spike in records completed where the survey taker answered, “Yes” to the question “Do you identify as Deaf?” While we wanted to be happy about this high response rate from a traditionally underrepresented group, we knew that we had not yet started our detailed outreach into local Deaf communities offering people the opportunity to participate in the survey study. This dissonance gave us reason to investigate response patterns within this pool of records more closely.

Indeed, we discovered hundreds of records whose answers to a combination of questions were all “yes” when in fact, it was highly unlikely to find hundreds of people with these shared characteristics. The questions we looked at were “Are you a Veteran or active member of the armed forces?,” “Do you identify as Amish or Mennonite?” and “Do you identify as Deaf?” We consulted with trusted advisors representing each of these communities separately. All three came to the same conclusion, that it was highly likely that these research entries were fake or spam.

As a result, we began the 8-month period of prevention and detection of suspicious records by creating a data integrity review team and focused heavily on monitoring the influx of records and our prevention strategies—a process we began just 2 days after we opened the study for recruitment. We recruited participants for 5 months and spent an additional 3 months actively amending and updating our detection efforts and checking in with community-engaged staff. During this time, we spontaneously developed inauthentic record infiltration prevention and detection processes that prioritized equity and inclusion of underrepresented populations. Our data integrity review team spontaneously developed prevention methods focused on ensuring that strategies utilized were as equitable as possible to maintain our overarching goal of recruiting historically marginalized populations.

To achieve this prioritization, we invited reflection on our initial observations of data inconsistencies leading us to suspect something unexpected and detrimental was happening. We continued to check in with community members who were already part of our community-based research process to double check that our filtering decisions were as equitable as possible. Ultimately, 3,861(19%) of the total 20,451 records in MHS 2022 were deemed to be authentic records. The following retroactive case report does not walk through specifics of how we moved from 20,451 to 3,861 records as those flags and processes are highly specific to the survey design and recruitment methods of My Health Story 2022. These operationalized details will not specifically translate to other projects. Instead, this report emphasizes the meaningful and translatable equity-focused elements of our spontaneously developed inauthentic record prevention and detection processes that were essential to protecting our primary focus of amplifying the inclusion of underrepresented populations in this project.

## 3 Inauthentic record prevention

### 3.1 Prior research

*S*trategies exist to help identify and mitigate inauthentic responses. Automated task or “bot-generated” technology is evolving at such a rate that existing strategies thought to be useful are already proving to be ineffective. Multiple research studies call attention to the ineffectiveness of identity validation techniques like ReCAPTCHA and survey screeners, making it difficult to protect against the infiltration of bot-generated survey responses in online survey research (3, 5, 20). Researchers experiencing these infiltrations also noted their surprise at how techniques used to catch or prevent spam were ultimately thwarted, indicating that human modifications to automated-task survey bots were likely being employed in an on-going fashion (21, 22). While we also included ReCAPTCHA and incorporated a content-based question into the beginning of the survey, it became clear early on that inauthentic survey takers were “learning” how to correctly move though these strategies.

Common strategies focus on identifying and removing spam through specific survey design considerations and survey-taking behavior checks, such as including honey pot questions, which are only readable by a bot program, monitoring for duplicate IP addresses, adding attention-check questions, and looking for outliers in the elapsed time between the start and completion of a survey (23). Honey pot questions, while generally effective, require programming expertise that public health department staff and community-based researchers may not have access to. Together, these strategies may provide multiple flags through which to identify inauthentic responses.

However, some of these strategies may unduly exclude authentic records among people who have reasonable and just reasons for triggering one or more of these flags. For example, removing records with duplicate IP addresses may unduly remove authentic records where multiple people from a household or who are using a public internet source, such as at a coffee shop or library, share an IP address and could all be legitimate survey records. Time-elapsed from survey start to survey completion could also vary greatly based on a number of factors. Inauthentic survey takers are not necessarily the fastest or slowest survey takers (24).

### 3.2 Evolving prevention strategies

Originally, the MHS 2022 survey did not have any protective features embedded in its design as our research team was unaware of the increased threat of inauthentic survey responses. All prevention and detection activities requiring amendments to our survey and recruitment strategies were IRB-approved prior to implementation. We continued to rely on data equity principles (14) as we recognized the need to pivot due to the infiltration of inauthentic data. We employed various strategies throughout data collection including the addition of reCAPTCHA, new REDCap survey links, adding security questions, removing incentives, adding geofencing, and conducting email validation. Our data integrity team continuously reflected on possible unintended impacts to inclusion of underrepresented populations as adjustments were made. Figure 2 summarizes the daily count of records overall and the timeline of preventative measures initiated during the data collection to keep inauthentic survey takers from accessing the survey. We elaborate on a few of the key successful and unsuccessful strategies for preventing and detecting inauthentic records.

**Figure 2:**
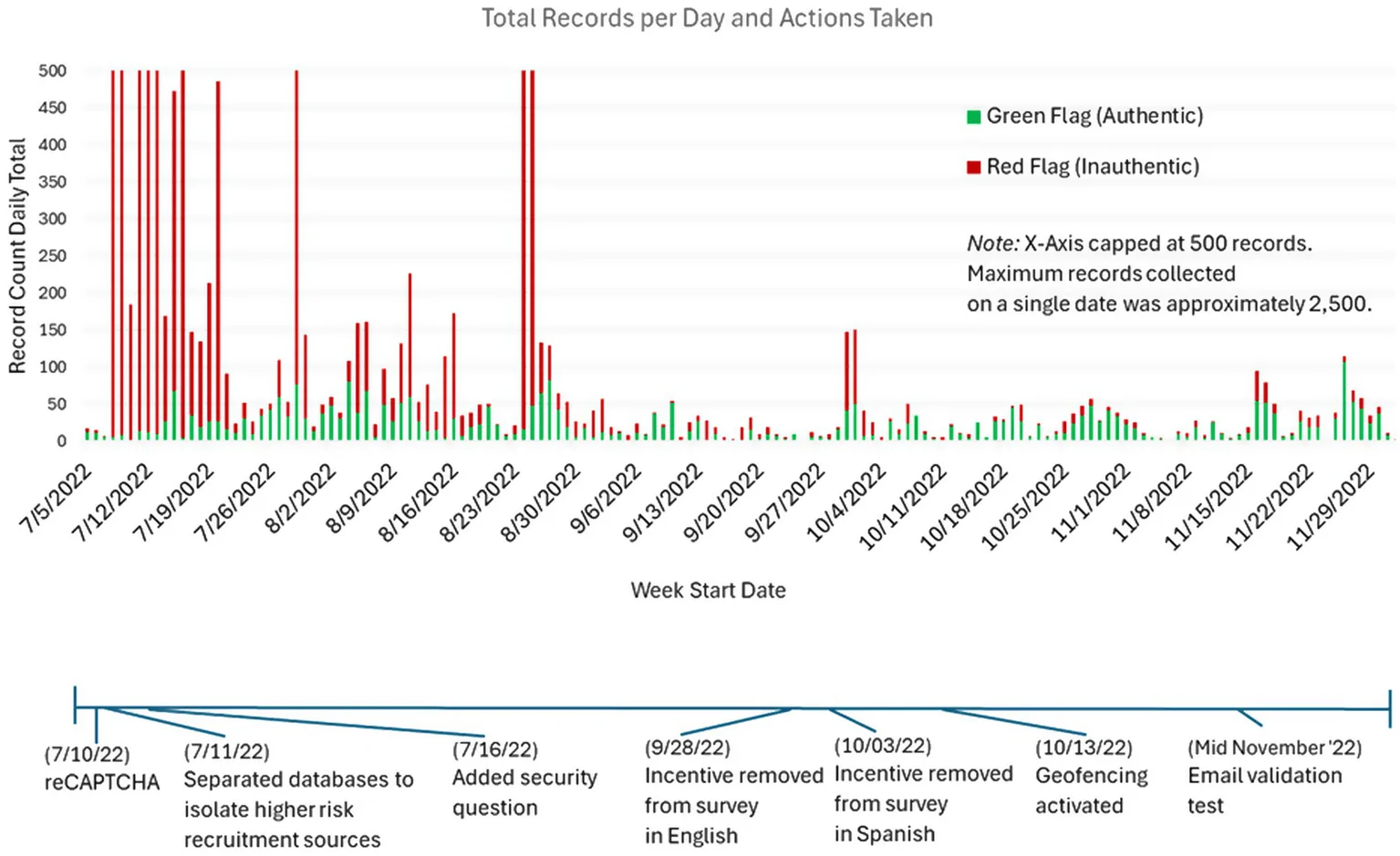
Actions taken to prevent influx of inauthentic records in My Health Story 2022.

To our surprise and subsequently corroborated in research reports (2, 4, 25), ReCAPTCHA was ineffective. We continued to see upwards of 1,000 or more new survey records per day, even after initiating ReCAPTCHA. When monitoring incoming data, we noticed improbable responses to key demographic questions that made us further question the authenticity of the completed surveys received. Large frequencies of unlikely combinations of “Yes” answers to specific questions like, “Are you Deaf?,” “Are you a Veteran?” and “Are you a member of an Amish or Mennonite community?” all in the same survey were suspicious and unlikely to be truthful responses. There were other oddities that we noted and tested through the 8-month prevention and detection journey.

We consulted with an IT-forensics expert and learned that our immediate reaction to inactivate and copy the survey along with linking new REDCap survey database website addresses to TinyURL for tracking was important. Spammers could easily find the relationships between the TinyURL links posted on our recruitment materials and the data collection server website addresses. Ultimately, the two most effective preventative actions were receiving IRB approval to remove the monetary incentive from our online survey and recruitment materials and installing a geofence around our entire REDCap server. The geofencing prevented all international IP-addresses from accessing the server where web-hosted survey links resided. However, due to the complexities of IP-address assignment across different devices, such as cell phones, tablets and computers, we were advised that it would be very difficult to further refine the geofence to a more specific geographic boundary.

As noted in Figure 2, we also added a security question that asked people to answer a question requiring knowledge about the catchment area to determine if a record was suspicious. With limited time to develop and test appropriate questions, we came up with one that allowed us to see if the answer to the question was possibly accurate. It became clear that the inauthentic survey takers had figured out an answer that would make sense and began using that same answer repeatedly, no matter which county they selected that they were from. This ultimately resulted in our abandonment of trying to find the most effective question as we did not have time to properly develop, test, and select a better set of security check questions.

Throughout these preventative action steps, our in-person ambassadors were still out in communities recruiting people to take the survey. Ultimately, about 50% of the total final record count for MHS 2022 was collected by the Ambassador team. These records were crucial to ultimately developing a set of additional post-hoc flags to detect and weed out suspicious records. Ambassador-collected data became one source of what we referred to as “green” or “trusted” data. In the following section, we describe additional ways we worked to detect suspicious records in the final months of recruitment and for 3 months after data collection ended.

## 4 Inauthentic record detection

After the geofence was in place and tested, we turned our attention to weeding out inauthentic survey responses from the data pool that was actively growing as data collection was still ongoing at this point. The data integrity team of research trained staff continuously met to review and monitor any impacts or unintended consequences on equity in inclusion of underrepresented populations. Ultimately, we developed a color-coded set of definitions based on survey content and survey taking behaviors. We looked for patterns in the data to operationalize flagging definitions. The data integrity team was charged with supporting the work of adapting the methods and definitions of color-coded detection flags while simultaneously reflecting on possible unintended consequences to inclusion of underrepresented populations.

The color coding began as a set of red and green definitions representing highly suspicious and trustworthy records, respectively. The TinyURL link tracked to the survey database allowed us to see specifically which recruitment tools were receiving the highest quantities of traffic. Although we were not able to directly match TinyURL web address traffic records with specific survey records, we were able to track the IP addresses of TinyURL website traffic and trace the timestamps to a range of timestamps on various REDCap records. Resulting from this, we were able to determine that the majority of suspicious activity was occurring through TinyURL links that were clicked from recruitment tools placed on the internet as the primary distribution point. The highest offenders were the TinyURL links located in paid advertisements on social media and public websites as well as the TinyURL links shared via social media posts to publicly accessible business pages and groups.

Additionally, we had full knowledge of when the TinyURL links were disconnected from REDCap survey databases. We used this information in the creation of some of the red-flag definitions. Ultimately, one of the easiest ways to identify inauthentic records was to mark records with a red flag that came into the inactive REDCap databases after the TinyURL links originally associated with those REDCap databases were deactivated. This was essential to the evolving set of color-coded flag definitions that we developed and refined with an equity-focused reflection process.

The color-coded flag definitions evolved over the entire 8-month period into a 4-color, 3-color, and back to 2-color set of flag definitions. The refinement of flag definitions was partially based on examining the percentage of records in the “green” data and the “suspicious data” with a particular flag or combination of flags. Then, we created a ratio showing us the odds that a flag was present in the “suspicious” group compared to the “green” group of records. This strategy gave us a clear understanding of which flags were better at calling out suspicious records. These flag definitions were largely based on operationalizing suspicious survey-taking behaviors, such as, flagging records with timestamps between 1 a.m. and 4 a.m. in our time zone.

We spent many meetings discussing the potential inclusion or exclusion of flags with odds relationships that were moderately sized or larger and we omitted the use of flag definitions that had the lowest odds of being present in “suspicious” data. To protect the integrity of our efforts and the application to future projects, we will not disclose details of the flag definitions here. Additionally, before testing for the presence of flags in either subset of data, we discussed and applied the principle of inclusion by asking ourselves, “Is it possible that an authentic record could be inadvertently flagged erroneously as an inauthentic record using these criteria?” We assert that these reflection questions themselves are focal process elements that promote equity in the detection and prevention of inauthentic records.

We also repeatedly consulted with community members for their opinions on various survey-taking behaviors and content anomalies that might warrant inclusion rather than automatic exclusion from the final dataset. In addition to developing definitions to identify inauthentic records, fundamental to our data-cleaning process was the ability to track “green” or “trusted” data records. We flagged as “trusted” any data records recruited by our in-person research ambassador team and marked them as “green” flagged data, barring any flags found through routine data cleaning, such as a high volume of skipped questions. To protect the future use and integrity of our flagging criteria, we intentionally omit examples of flag definitions in this paper. Instead, we encourage future online survey researchers to examine their recruitment methods for possible risk of infiltration of inauthentic records. Additionally, we assert that, separately from traditional data cleaning methods, time and resources should be reserved for equity-focused iterative reflection and the development of project specific-flags and techniques for the prevention and detection of inauthentic records.

## 5 Recommendations

Table 2 summarizes our recommendations for equitable spam prevention and detection in online, anonymous public health surveys. MHS 2022 prevention and detection processes benefited from the multimodal in-person and internet-based recruitment strategies already built into the study design. To date, practices for preventing and identifying inauthentic records do not consistently prioritize mitigating potential biases to inclusion of underrepresented research populations introduced by logic check-based record flagging algorithms (4, 5). It is important that researchers include a pre-designed plan for inauthentic record prevention and detection in collaboration with members of the target research population to ensure that flag definitions and procedures are inclusive and accessible. Multi-modal recruitment plans that include in-person and internet-based recruitment strategies help to ensure that data cleaning and flagging protocols remain accessible, inclusive, and respectful to a broad range of survey takers.

**Table 2:** Summary of prevention, detection, and regulatory recommendations in My Health Story 2022.

*Prevention*
Use a geofence surrounding data collection software to prohibit IP addresses from outside the project catchment area from accessing the survey.
Protect staff time and budget resources for a data integrity forensic efforts and potential programming of more complex software programming protections, such as “honey pot” questions.
Plan ahead to create and test logic or attention-check questions within your target population prior to initiating study recruitment.
Do not mention the presence of incentives on recruitment materials and do not automate the study payment process upon completion of the project survey.
*Detection*
Create a data integrity review team to mitigate potential unintended exclusion of underrepresented populations.
Use multi-modal recruitment methods and track data collected via in-person and online recruitment tools, separately.
Create separate survey data collection databases for records stemming from online recruitment methods and for those stemming from in-person, or more trusted, recruitment methods.
Create process and content-based flag definitions and repeatedly test them in records that are trusted verses suspicious before deciding if they are effective and reliable.
Review any flag definitions with target population members to check for biases and potential unintended, overly exclusionary consequences.
*Regulatory*
Include language in the general information section, or informed consent, indicating the potential risk that unintended, inauthentic record infiltration could compromise the integrity of the dataset.
Include language indicating that study incentives may be delayed or cancelled if a participant record is flagged as inauthentic.

There are also equity implications of inauthentic record infiltration that impact research investigators’ ability to meet the requirements set forth by 45 CFR 46 (11). According to Section 46.11 of federal regulation policy 45 CFR 46 governing human subjects research, expedited research projects must meet criteria ensuring that “Selection of subjects is equitable.” (11) Methods to detect and prevent inauthentic record infiltration may unfairly limit the inclusion of some populations in anonymous online survey studies. We recommend that researchers doing online survey data collection include a sentence in their introductory summary informing potential survey takers of the chance that inauthentic record infiltration might occur and could cause the entire dataset to be unusable. We also strongly recommend that researchers plan ahead to include a data equity review team focused on mitigating any possible unintended negative consequences of inauthentic data detection strategies.

Regarding prevention, we strongly recommend that investigators do not indicate that there is an opportunity for compensation or incentives on recruitment tools. While we do not know the specific motivations behind inauthentic record takers, we do know that we saw a decrease in inauthentic record infiltration after we removed the mention of an incentive on our recruitment flyers and social media messages. Additionally, we know that the geofence we constructed did reduce the number of pings coming from areas way outside of the catchment area for our project. We recommend that a digital geofence be placed around the installed survey software to limit the exposure of the survey database to ineligible respondents. Lastly, we highly recommend a mixed approach to recruitment that includes tracking data collected in-person and online. Maintaining a source of “green flag” records, or those with the lowest likelihood of being inauthentic, was a key element of our overall inauthentic record detection process.

## 6 Conclusion

Inauthentic records jeopardize the quality and reliability of public health surveys (3, 5). Specifically addressing removal of inauthentic records takes a large amount of time and resources beyond what is required for traditional data cleaning (3). Investigators can lower the risk that infiltration results in unusable data by planning ahead and reviewing the latest strategies for dealing with continuously evolving efforts to thwart well-intentioned research efforts (3, 21, 22). Ultimately, the prioritization of preventing, finding, and removing inauthentic records thwarted our pre-planned efforts to proactively recruit using quota-based sampling across multiple categories of population demographics. Had we not recognized the infiltration, our resulting analyses would have been flawed and inaccurate.

Our findings pertaining to mean age and ZIP Code proportions are not meant to be generalizable, but to provide evidence of how an unexpected event that occurred during data collection, if left unaddressed, would have impacted two of the data points we would have checked in our analysis. There are no detection or prevention efforts that will work for all scenarios. Our experience demonstrates the importance of prioritizing resources toward equitable prevention and detection planning before collecting data. In sharing our journey, we hope to help other researchers maintain their focus on equity in recruitment of underrepresented populations, while protecting the integrity of the project through decreasing the likelihood that inauthentic record infiltration will irreversibly thwart the interpretability of dataset findings.

The creation of flag definitions is highly contextual to the design, content, and recruitment methods of a research project. We encourage researchers to develop specific plans to equitably detect and prevent infiltration of inauthentic records, prior to beginning data collection. To protect the integrity of flag definitions for future use, we do not present explicit definitions. The authors encourage interested readers to contact them for more detailed information about flag definitions.

## Data Availability

The raw data supporting the conclusions of this article will be made available by the authors, without undue reservation.
